# Retrograde type a dissection in a 24th gestational week pregnant patient – the importance of interdisciplinary interaction to a successful outcome

**DOI:** 10.1186/s13019-018-0724-7

**Published:** 2018-05-02

**Authors:** Jerry Easo, Michael Horst, Bernhard Schmuck, Rohit Philip Thomas, Steffen Saupe, Malte Book, Alexander Weymann

**Affiliations:** 1 Department of Cardiac Surgery, European Medical School Oldenburg-Groningen, University Hospital Oldenburg, Carl von Ossietzky University Oldenburg, Rahel Straus Str. 10, 26133 Oldenburg, Germany; 2 Department of Diagnostic and Interventional Radiology, European Medical School Oldenburg-Groningen, University Hospital Oldenburg, Carl von Ossietzky University Oldenburg, Oldenburg, Germany; 3 University Department of Obstetrics and Gynaecology, European Medical School Oldenburg-Groningen, University Hospital Oldenburg, Carl von Ossietzky University Oldenburg, Oldenburg, Germany; 4 Department of Anaesthesiology, Critical Care, Emergency Medicine and Pain Therapy, European Medical School Oldenburg-Groningen, University Hospital Oldenburg, Carl von Ossietzky University Oldenburg, Oldenburg, Germany

**Keywords:** Aortic dissection, Pregnancy, Stent-grafting

## Abstract

**Background:**

Type A Dissection in pregnancy is a devastating medical condition with 2 lives at stake and unclear strategy at early gestational stages. We describe a successful outcome, clearly dependent on the coordination of all involved disciplines.

**Case presentation:**

This case history describes a 28 year old female with a 24th week pregnancy gravida 2 para 0 with a DeBakey Type I aortic dissection, diagnosed via ultrasound. Surgery was perfomed on the day of diagnosis. After conferral with the mother, caesarean section was performed and a 690 g fetus could be delivered and was immediately transferred to the neonatal unit. Subsequent aortic repair was performed after hysterectomy, with replacement of the ascending aorta and hemiarch treatment. Intraoperatively no entry in the ascending aorta or transverse arch could be demonstrated, so that a retrograde Type A with entry distal to the left subclavian had to be postulated. We decided to perform subsequent computer tomography, demonstrating multiple entry sites in the descending aorta distal to the left subclavian artery. Successful endovascular treatment could be performed with a Medtronic Valiant Stent via a transfemoral approach. The further hospital stay was uneventful and the patient could be discharged on the 18th postoperative day. The baby demonstrated fighter qualities and could be discharged home after a 3 month hospital stay to be reunited with his mother.

**Conclusion:**

Prompt diagnosis, precise coordination between all involved subspecialties and ultimately, as in this case, definitive treatment in consensus with operative and interventional departments have led to a successful outcome and encourages us in our daily struggle in this often demanding surgery.

## Background

Aortic dissection remains a devastating medical condition with a prevalence of estimated 2.9 per 100,000 person years [1], associated mainly with arterial hypertension and often related to connective tissue disorders such as Marfan’s Syndrome or Ehlers Danlos Syndrome. In pregnancy, acute aortic type A dissection has an overall incidence of 0.4 per 100,000 person years, often presenting in the third trimester attributed to hemodynamic alterations taking place in late pregnancy. In woman under 40 years of age half of all aortic dissections occur during pregnancy or during the peri-partum period [2]. Gestational hypertension, pre-eclampsia and hormonal changes during pregnancy may predispose to aortic dissection, in addition high levels of oestrogen and progesterone may cause degeneration of the aortic wall, similar to cystic medial necrosis [3–8]. Early diagnosis and successful coordination of care between the cardiothoracic surgeon, anaesthesiology, interventional radiologist, obstetrician and paediatrician is mandatory for successful outcome of such complex patients.

## Case presentation

**Fig. 1:**
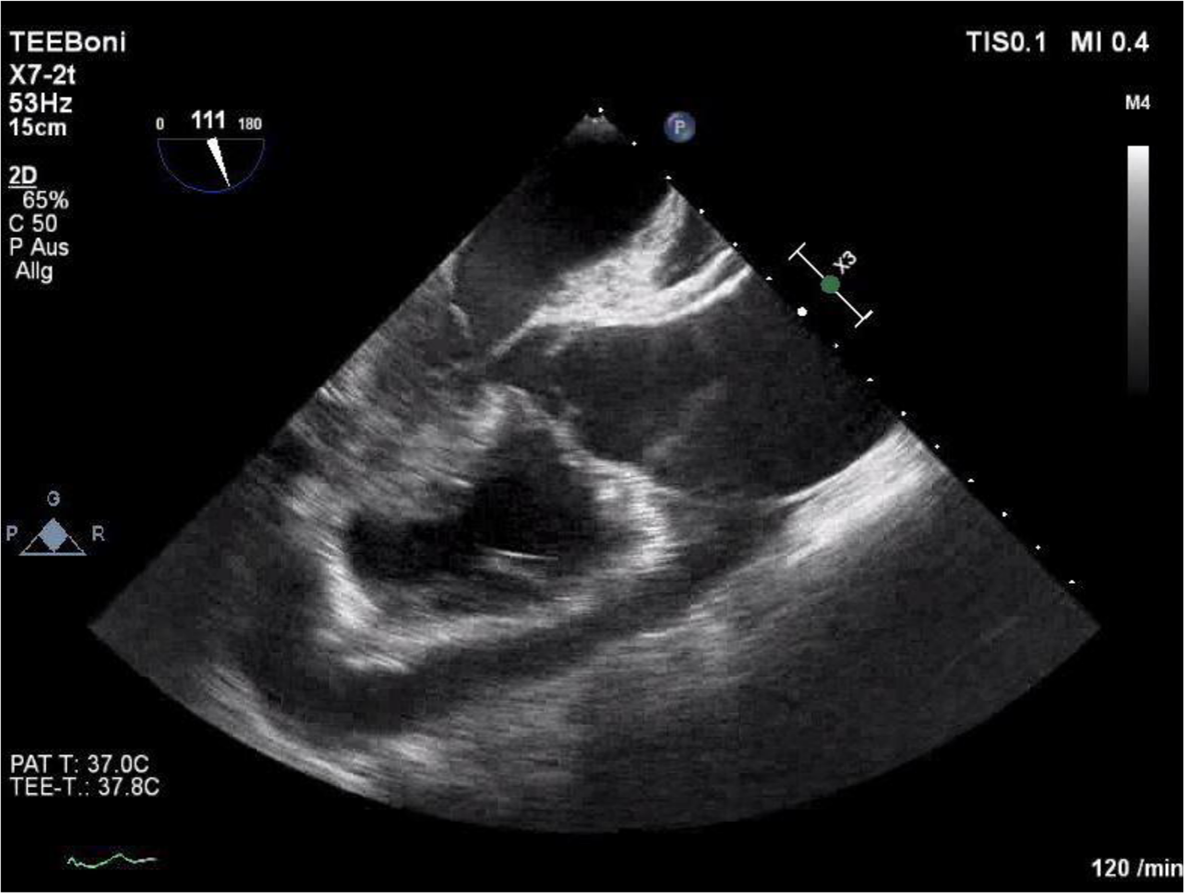
Transoesophageal echocardiography demonstrating pericardial effusion and dissection

A 28 year old woman with a 24th -week intrauterine pregnancy gravida 2 para 0 was admitted to the emergency department of the referring hospital with chest and back pain. Ultrasound diagnostic demonstrated pericardial effusion and a dissection membrane in the ascending and descending aorta (Fig. 1). The patient was immediately transferred with the diagnosis of a DeBakey Type I Aortic dissection. Further comorbidities included arterial hypertension, renal insufficiency stage 4 with a history of kidney transplant 2014 after preeclampsia after childbirth 2007, among others.. Initial consensus was found to minimise the operative risk for the mother and to perform surgery leaving the fetus in vivo and choosing an optimal operative strategy with a mild hypothermia and arch inspection to limit the period of circulatory arrest to an absolute minimum. After conferring with the pregnant mother however, she decided to deliver the baby via caesarean section despite the early phase of prior to aortic repair. To minimise the perioperative risk of bleeding the patient consented to an operative hysterectomy before heparinisation for the establishment of extracorporal circulation.

Surgery was performed on the day of diagnosis in a collaborative manner, with induction of general anaesthesia immediately prior to caesarean section performed via a Misgav Ladach operative technique and subsequent supracervical hysterectomy. The fetus was immediately transferred to the neonatal paediatricians, the initial body weight was 690 g with an APGAR 2/4/6 and a naval pH of 7.19.. After semi-closure of the abdomen we proceeded with the surgical treatment of the aortic dissection. Arterial cannulation was established via the right subclavian artery, mild hypothermia was targeted and exposition of the aorta and supraaortic vessels was achieved. Aortotomy demonstrated the dissection reaching into the acoronary sinus, the right and left coronary sinus showed no signs of dissection. After resection of the dissected aorta to the sinotubular ridge and application of Bioglue® (Cryolife Inc. Kennesaw, U.S.A) for stabilisation of the dissected noncoronary Sinus a pledget strip stabilised supracoronary ascending aortic replacement using a 28 mm Vascutek Gelweave vascular prosthesis was performed. After reaching the aimed 28 °C temperature, circulatory arrest was established and selective antegrade cerebral perfusion was administered over the brachiocephalic trunk and the left common carotid artery. The left subclavian artery was snared down by tape to reduce subclavian steal. Inspection of the aortic arch showed however no entry site in the ascending aorta or the transverse arch, even distal to the left subclavian artery as far as visible. A possible retrograde type A dissection had to be postulated as possible cause of the aortic pathology. A rapid decision had to be made as to whether to extensify the procedure in form of a frozen elephant trunk procedure to occlude possible entry sites in the descending aorta, or to secondarily visualise the tears of the descending aorta via computed tomography and secondary treatment of the underlying pathology. The less radical approach was chosen and a hemi-arch replacement was performed in a circulatory arrest of 21 min using SACP for 12 min, INVOS® (Somanetics, U.S.A) monitoring showed no hemispheral differences in saturation. Postoperative echocardiographic examination demonstrated trivial aortic regurgitation. After adequate haemostasis was achieved the gynaecologists were contacted for definitive closure of the abdomen.

**Fig. 2:**
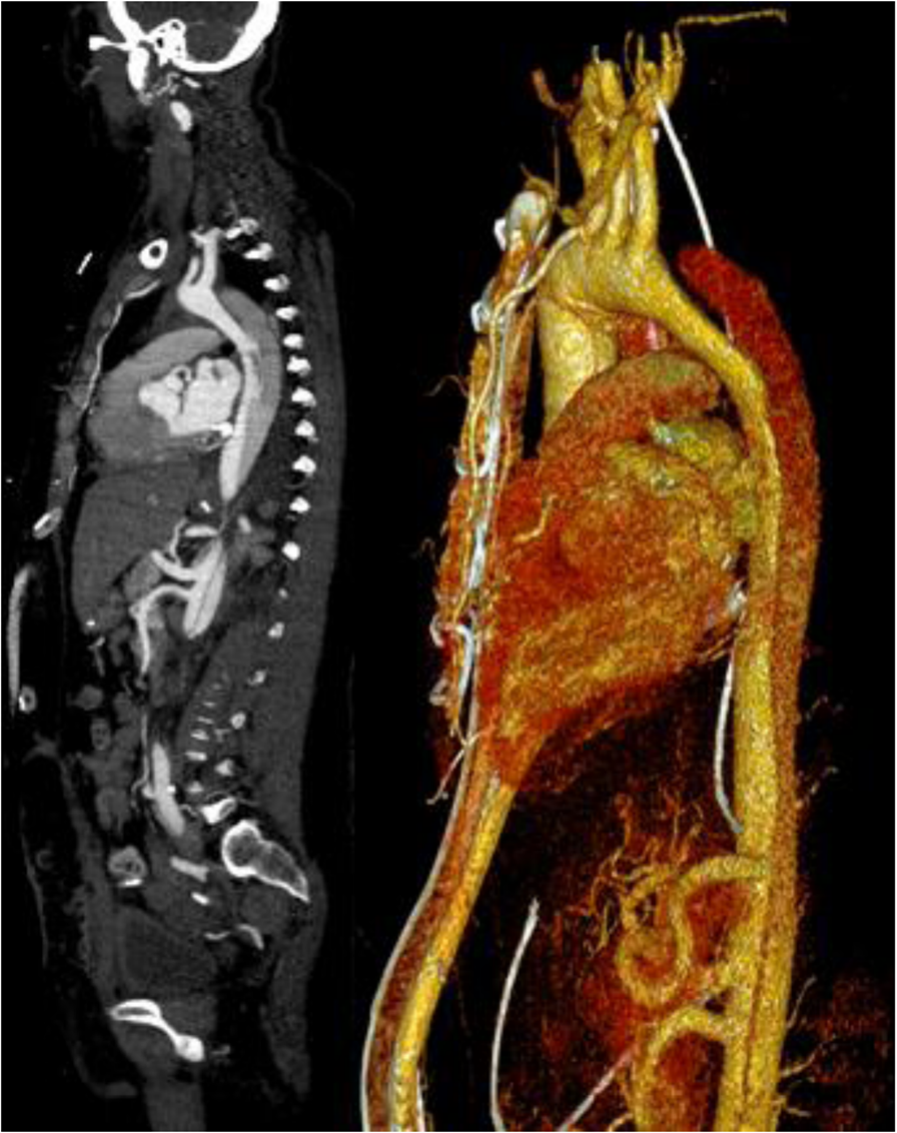
CT scan demonstrating entry site distal to the left subclavian artery

**Fig. 3:**
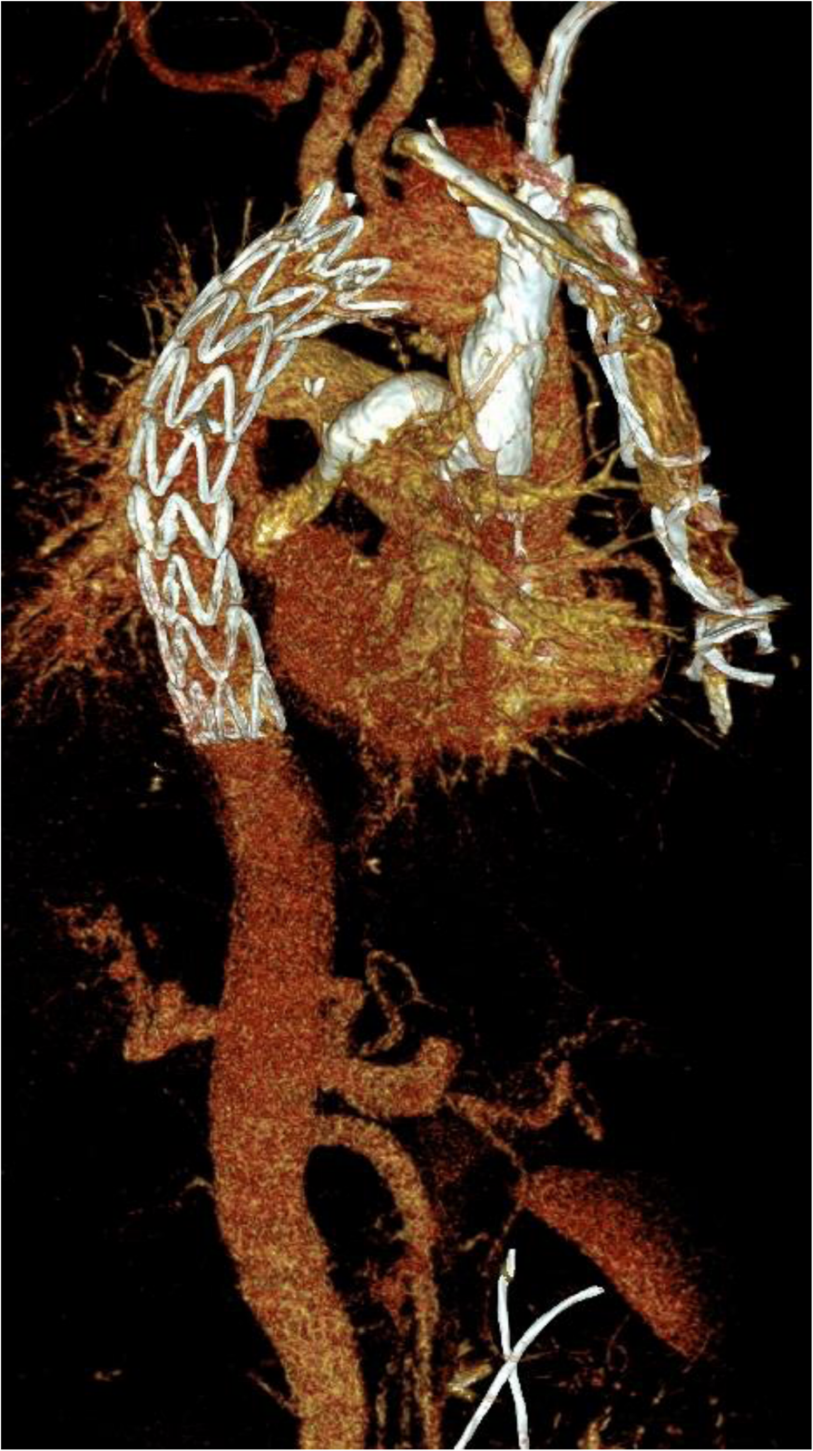
Postinterventional CT scan after deployment of stentgraft

The initial postoperative period showed no complications and subsequent computer tomography of the aorta was performed. This demonstrated multiple entry sites in the descending aorta approximately 6 cm distal the left subclavian artery, with adequate perfusion of the visceral arteries and a narrowing of the true lumen following the origin of the left subclavian artery (Fig. 2). The transplanted kidney was anastomosed to the left common iliac artery with adequate perfusion. The decision was quickly made to treat the descending aorta via an endovascular stentgraft to obliterate possible retrograde perfusion to the aortic arch. Under general anaesthesia percutaneous access to the right common femoral artery was achieved. After pre-placement of sutures of Prostar XL® closure device (Abbott Vascular, Santa Clara, CA, USA), a 10F sheath was introduced and angiograms of the thoracic and abdominal aorta were performed with a pigtail catheter. A super-stiff guide wire (Back-up Meier, Boston Scientific Corporation, Marlborough, MA, USA) introduced through the pigtail catheter ensured the correct position of the guide wire in the true lumen. Consequently a Valiant Captivia thoracic stent graft (32/32/157 mm, 22F, Medtronic) system was introduced into the aortic arch and implanted in such a fashion that the covered part of the stentgraft started just distal to the origin of the left subclavian artery. The control angiogram showed adequate sealing of the descending thoracic aorta and improved flow to the true lumen of abdominal aorta (Fig. 3). The control CT examination 5 days later showed improved flow in the true lumen and abdominal. Furthermore no dissection could be demonstrated in the aortic arch.

Further hospital stay was uneventful without neurological sequelae and the patient could be discharged to the rehabilitation centre on the 18th postoperative day. The baby could be definitively extubated on the 24th postoperative day, he required phototherapy due to hyperbilirubinemia, the patent ductus could be successfully treated by Ibuprofen and the baby could be discharged home safely with a weight of 2015 g and 43 cm height after a 3 month hospital stay.

## Discussion

Aortic dissection in woman under 40 years of age are associated with pregnancy in 50% of patients, often related to hormonal changes with high levels of oestrogen and progesterone as well as risk factors predisposing to aortic stress such as systemic arterial hypertension, most often in the third trimester of pregnancy [2]. Tachycardia, increased cardiac output observed during pregnancy, expanded intravascular volume and position dependent compression of the distal aorta and iliac arteries by the gravid uterus stresses the proximal aorta [4]. Preeclampsia is another risk factor often associated with aortic dissection [9]. In most cases of aortic dissection the histological findings show medial degeneration with mucoid degeneration and loss of elastic fibres. Acute type A aortic dissection require emergency surgery, with restoration of blood flow, with mortality rate of untreated dissection increasing by 1-3% per hour after presentation [10].

Aortic dissection in pregnancy poses several problems in the therapeutic strategy, with understandable reluctance to perform immediate repair. In many cases described the child was delivered first and treatment of the aortic dissection was performed thereafter, usually by standard treatment of the ascending aorta and open distal anastomosis in circulatory arrest. These described cases most often present the fetus in a viable condition with a gestational age past the 34th week. Based on the experience of Zeebregts et al. following guidelines were proposed: In a pregnant patient with an acute aortic type A dissection therapy should be aimed at saving 2 lives, determined by the gestational age of the fetus. Before 28 weeks of gestation, aortic repair with the fetus kept in situ is recommended, if the fetus is viable after 32 weeks of gestation primary caesarean section followed by aortic repair is the treatment of choice [11]. These recommendations are shared by a large series by Zhu et al., prioritising maternal survival [12]. High flow high pressure cardiopulmonary bypass is recommended as safest for the fetus, moderate hemodilution (haematocrit of more than 25%) is aimed for [13]. Due to induction of fetal bradycardia by hypothermia, systemic hypothermia should be avoided, rewarming may also lead to premature intrauterine contractions. Open distal repair, which is preferable, should be avoided [14].

The patient described in this report suffered pre-eclampsia during the first pregnancy, which led to renal insufficiency with consecutive kidney transplant in 2014, further comorbidities included arterial hypertension, both predisposing factors to aortic dissection. Due to the early gestational period of the 24th week optimal planning was complicated, keeping the fetus in situ as recommended would have possibly led to the demise of the fetus Due to unclear dissection pathologies of the transverse aortic arch it was not possible to avoid circulatory arrest as arch inspection was necessary to rule out possible entry sites in this segment of the aorta. We recommended, as per guidelines, this form of treatment,ultimately the mother decided on delivery of the fetus per caesarean section despite the possible detrimental consequences for the baby due to the early stages of the pregnancy.

The intraoperative suspicion of a retrograde Type A dissection after opening of the aortic arch, confirmed subsequently by CT examination, led to a dilemna for choice of optimal treatment. Antegrade stent-grafting of the descending aorta and total arch replacement would have been possible, the risk of longer circulatory arrest and their adjunct detrimental consequences had to be weighed against hemiarch replacement and subsequent stent-grafting after visualisation of the entry tears. The successful outcome using this treatment algorithm encourages us, other forms of treatment such as use of the frozen elephant trunk technique however may have led to a similar positive outcome.

The optimal interaction between cardiovascular surgeon, anaesthesiologist, paediatrician, gynaecologist and interventional radiologist was imminently important for the successful treatment of the patient. Preoperative CT scanning of the aorta would have provided valuable information for the surgical planning of treatment, however this was not possible without radiation risk and was avoided as the decision for operation was not dependent on the radiologic findings. Echocardiography diagnosed the dissection in this case, with described sensitivity and specificity up to 75 and 90% respectively. Transoesophageal echocardiography overcomes many of the limitations of transthoracic echocardiography and show sensitivity and specificity data as high as 99% and 98 respectively [15]. Intraoperative transoesophageal echocardiography was performed and entry tears were not clearly seen in the descending aorta so that the subsequent CT examination provided the necessary information for optimal treatment.

The decision of the mother to deliver the baby preoperatively was, retrospectively seen, a correct decision. The baby developed well over a 3 month hospital stay despite all possible detrimental consequences associated with such a premature birth. Clearly the excellent work of the neonatal paediatrician and nursing contributed to the successful outcome.

The diagnosis of aortic dissection is often overlooked, with misdiagnosis occurring in 85% of patients presenting with acute dissection [1]. Many case reports describe treatment of aortic dissection well postpartum due to missing of initial diagnosis. Awareness of this rare medical condition and prompt diagnosis of the referring centre was surely an optimal prerequisite for the successful outcome of this complex case.

## Conclusion

Treatment of acute type A aortic dissection in pregnant patients at such an early gestational period with the first 24 weeks of pregnancy poses a grave dilemma for the optimal therapeutic strategy. Prompt diagnosis, precise coordination between all involved subspecialties and ultimately, as in this case, definitive treatment in consensus with operative and interventional departments have led to a successful outcome and encourages us in our daily struggle in this often demanding surgery.

## Abbreviations

APGAR: Activity, Pulse, Grimace, Appearance and Respiration Score
INVOS: Cerebral oximetry monitoring
pH: Power of Hydrogen
SACP: Selective antegrade cerebral perfusion
